# Functional Cognitive Disorder as a Cause of Cognitive Impairment Not Linked to Dementia

**DOI:** 10.1192/bjo.2026.11879

**Published:** 2026-06-30

**Authors:** Aniruddha Rajkonwar

**Affiliations:** Royal College of Psychiatrists, Malton, United Kingdom

## Abstract

**Aims:**

Background-Functional Cognitive Disorder ( FCD) is an under-recognised condition which is different from dementia or Mild Cognitive Impairment. It causes problems with memory but unlike dementia, it is not a neuro-degenerative condition.

**Methods:**

Case Report- 63 year old female presenting with cognitive problems, corroborated with a collateral history and scoring low 56/100 in Addenbrooke cognitive examination. Higher cortical functions processing, judgement, awareness of risks and orientation and functioning intact. CT scan was unremarkable and no structural changes related to dementia seen. The clinical presentation was incongruent with the low scores in the Addenbrooke. Patient recently diagnosed with functional neurological disorder as she had been presenting with tremors.

**Results:**

Discussion- Functional cognitive disorder (FCD) refers to complaints of persistent problematic cognitive difficulties, when accompanied by positive features termed ‘internal inconsistency’, and which are not better explained by another disorder e.g. a neurodegenerative disease process. Internal inconsistency is when a person may present with significantly impaired ability to perform tasks, particularly when the task is the focus of attention. Therefore, the individual components required to execute the task are intact, but there is difficulty engaging them at the appropriate intensity or duration on demand.

**Conclusion:**

Conclusion- Although uncommon, functional cognitive disorder should be considered as a differential diagnosis in people presenting with cognitive difficulties where the clinical picture does not fit the picture of a neuro-degenerative condition like dementia. A misdiagnosis as dementia will a profound impact on a person’s personal, social and occupational aspects of life. The right diagnosis can provide re-assurance to people and enable them to be sign-posted to the right support services. This can reduce distress, stop people from being sent for unnecessary investigations, being misdiagnosed as a dementia and enable them to access the appropriate help for their symptoms.

